# The modality-switch effect: visually and aurally presented prime sentences activate our senses

**DOI:** 10.3389/fpsyg.2015.01668

**Published:** 2015-10-30

**Authors:** Elisa Scerrati, Giulia Baroni, Anna M. Borghi, Renata Galatolo, Luisa Lugli, Roberto Nicoletti

**Affiliations:** ^1^Department of Philosophy and Communication, University of BolognaBologna, Italy; ^2^Department of Psychology, University of BolognaBologna, Italy; ^3^Institute of Cognitive Sciences and Technologies, Italian National Research CouncilRome, Italy

**Keywords:** modality-switch effect, property verification, priming paradigm, embodiment, grounded theories of concepts

## Abstract

Verifying different sensory modality properties for concepts results in a processing cost known as the modality-switch effect. It has been argued that this cognitive cost is the result of a perceptual simulation. This paper extends this argument and reports an experiment investigating whether the effect is the result of an activation of sensory information which can also be triggered by perceptual linguistically described stimuli. Participants were first exposed to a prime sentence describing a light or a sound’s perceptual property (e.g., “The light is flickering”, “The sound is echoing”), then required to perform a property-verification task on a target sentence (e.g., “Butter is yellowish”, “Leaves rustle”). The content modalities of the prime and target sentences could be compatible (i.e., in the same modality: e.g., visual–visual) or not (i.e., in different modalities). Crucially, we manipulated the stimuli’s presentation modality such that half of the participants was faced with written sentences while the other half was faced with aurally presented sentences. Results show a cost when two different modalities alternate, compared to when the same modality is repeated with both visual and aural stimuli presentations. This result supports the embodied and grounded cognition view which claims that conceptual knowledge is grounded into the perceptual system. Specifically, this evidence suggests that sensory modalities can be pre-activated through the simulation of either read or listened linguistic stimuli describing visual or acoustic perceptual properties.

## Introduction

Object’s properties can be perceived through different sensory modalities. Thus, while detecting the color of a traffic light in a cross-road mainly involves the visual modality, perceiving the melody of a violin during a classic concert mainly involves the auditory modality. According to grounded theories of knowledge (Barsalou, 2008; for a recent discussion see Borghi and Caruana, 2015; Pecher and Zeelenberg, 2015), sensory information is also active when we process the concepts *TRAFFIC LIGHT* and *VIOLIN*^1^. In other words, processing concepts would imply a re-enactment of previously recorded and integrated perceptual information concerning the objects or entities they refer to. Hence, a similar pattern of neural activation in sensory systems would be preserved in representation: while processing the concept *VIOLIN*, for instance, the auditory system would re-enact states associated with hearing its sound. This re-enactment is also known as perceptual simulation.

According to embodied and grounded theories (see also Glenberg, 1997; Barsalou, 1999, 2003, 2009), the re-enactment evoked by linguistic stimuli represents a form of simulated experience. It is worth mentioning that the notion of simulation varies in detail and depth (Borghi and Cimatti, 2010; for a review see Decety and Grèzes, 2006). More specifically two slightly different views are taken into account in the Embodied Cognition theories. According to the first, the notion of simulation is mainly based on the re-enactment of past sensorimotor experience (Barsalou, 1999). The second view stresses the predictive aspect of simulation, suggesting that the automatic simulated re-enactment of the observed actions and objects is at the basis of a direct form of action preparation and comprehension (e.g., Gallese, 2009). Here we mainly focus on simulation as a form of multimodal re-enactment of previously sensory experiences.

A growing number of neuroimaging studies show that modality-specific brain areas are active during conceptual processing (for reviews, see Martin, 2001, 2007; Martin and Chao, 2001). For instance, when people process color names (e.g., *YELLOW*), color areas in the visual cortex become active (Simmons et al., 2007). Conversely, when people process concepts for which the auditory modality is important (e.g., *TELEPHONE*), auditory areas become activated (Kiefer et al., 2008). These results are consistent with the claim that people simulate concepts in sensory systems.

The behavioral literature offers further evidence in support of the assumption that perceptual information is engaged in conceptual processing, showing a cost for performance in terms of speed and accuracy when two different modalities alternate, compared to when the same modality is presented (Pecher et al., 2003, 2004; Marques, 2006; Vermeulen et al., 2007; van Dantzig et al., 2008). This effect, known as the modality-shifting effect or modality-switch effect (henceforth, MSE) was initially found in a pure perceptual study by Spence et al. (2001). Participants were faced with a visual, tactile, or auditory signal that could appear on the left or on the right. Their task was to detect the location of the signal (i.e., left or right) as rapidly as possible by pressing one of two pedals. Performance was faster and more accurate for trials that were preceded by a same-modality trial (e.g., visual–visual) than for trials that were preceded by a different-modality trial (e.g., auditory-visual).

Crucially, the MSE was replicated using a conceptual task (Pecher et al., 2003). Pecher et al. (2003) used a property verification task (see Collins and Quillian, 1969; Conrad, 1972; Smith et al., 1974; Glass and Holyoak, 1975; Rosch and Mervis, 1975; Solomon and Barsalou, 2001, 2004): participants were presented with short sentences having a ‘concept can be property’ scheme (e.g., ‘*BANANA* can be *yellow’*) and had to verify whether the property was true of the concept. Related pairs of property verification sentences alternated throughout the task: a context sentence (i.e., the one presented first) was always followed by a target sentence. Properties in both context and target sentences could be in one of six modalities (vision, audition, taste, smell, touch, and action). The key manipulation consisted in the fact that each target sentence could be preceded by a sentence with a property in the same or in a different modality. Results showed that properties were verified faster and more accurately in same-modality trials than in different-modality trials. For instance, participants were faster and more accurate when verifying the property *pastel* for *BABY CLOTHES*, if they previously verified the property *yellow* for *BANANA* (both visual) rather than the property *rustling* for *LEAVES* (auditory context – visual target). This finding suggests that conceptual processing strongly relies on perceptual and motor information.

However, two possible criticisms of the study by Pecher et al. (2003) lay on the fact that (1) their property verification paradigm might have involved less automatic processes compared to those that a simulation would entail (on the automaticity of simulation see Pulvermüller, 2005; Jeannerod, 2006); (2) the MSE with conceptual representations could be explained assuming that concepts are abstract, amodal symbols rather than grounded in perception and action systems (see Mahon and Caramazza, 2008 for a discussion).

As to the first criticism, indeed, it has been argued that simulations are fast, implicit and automatic and only involve exogenous attention. In a recent study, Connell and Lynott (2012) linked perceptual attention to conceptual processing (on the relationship between concepts and attention see also Myachykov et al., 2013). These authors claimed that while exogenous attentional mechanisms are involved when incoming stimuli automatically grab attention, endogenous attentional mechanisms are involved when people consciously focus attention on a particular modality (see also Connell and Lynott, 2010). Thus, only exogenous attentional mechanisms would be at work during a perceptual simulation, inducing, for instance, the automatic pre-activation of specific sensory modalities during reading. Automatically pre-activated specific modalities could then interfere with or facilitate the subsequent processing of semantic information yielding the MSE (see also Connell and Lynott, 2014). However, Pecher et al. (2003) had participants performing a double property verification task on each trial, one on the context and one on the target sentence. In addition, no time limit was provided to carry out the task^2^. Therefore, participants were possibly lead to rely on strategic processes involving endogenous attention, such as constructing a mental image of the concept and property described in the sentences. Although mental imagery can be considered as “the best known case of [ ] simulation mechanisms, [it] typically results from deliberate attempts to construct conscious representations in working memory, [whereas] other forms of simulation often appear to become active automatically and unconsciously outside working memory” (Barsalou, 2008, p. 619, see also Kiefer and Pulvermüller, 2012; Kiefer and Barsalou, 2013). For instance, Pulvermüller et al. (2000) showed that semantic activation in the sensorimotor cortex in passive reading tasks was present ∼ 200 ms after word onset which would reflect stimulus-triggered early lexico-semantic processes (i.e., simulation) rather than post-lexical processes (i.e., imagery, see also Pulvermüller, 2005; on the generation of mental images see Farah et al., 1989). Since in Pecher et al.’s (2003) paradigm participants had to perform a property verification task also on the context sentence and each sentence was presented until a response was given, one could reasonably argue that post-lexical processes involving endogenous attentional mechanisms could explain the MSE.

As to the second criticism, van Dantzig et al. (2008) sought evidence for the involvement of sensory information in conceptual processing that could not be explained by amodal symbols. According to amodal symbols accounts of concepts (Collins and Quillian, 1969; Smith and Medin, 1981; Barsalou and Hale, 1993), modal representations are turned into abstract, amodal symbols that represent knowledge about experience. Although being amodal, these symbols might still be organized so that to reflect their modality. The MSE with conceptual representations (Pecher et al., 2003) could hence hinge on connections between these symbols. van Dantzig et al. (2008) investigated the effect of a perceptual task such that of Spence and colleagues on a conceptual task such that of Pecher et al. (2003). More specifically, the authors asked participants to perform a perceptual left/right spatial discrimination task followed by a conceptual property verification task, with the latter used as the target task. On each trial, participants first detected left/right visual, auditory or tactile signals (i.e., a light flash, a tone or a vibration), as in Spence et al. (2001), and then judged whether a visual, auditory or tactile property was true of a concept, as in Pecher et al. (2003). Results indicate that participants were faster at verifying whether a property was true of a concept if that property was in the same sensory modality as the immediately preceding perceptual signal. Hence, participants, for example, were faster at verifying that *BABY CLOTHES* are *pastel* if they previously detected a light flash rather than a tone or a vibration. This finding provides evidence that pure perceptual processing (i.e., perceiving stimuli without any semantic meaning) affects the activation of conceptual processing. Since no meaningful relationship existed between the perceptual signals of the first task and the concepts of the second task the authors could conclude that the MSE cannot be explained by amodal symbols.

The present study aims at investigating whether the MSE is the result of the activation of sensory information when exogenous attentional mechanisms are involved. To this end, we introduced two key modifications of Pecher et al. (2003) and van Dantzig et al.’s (2008) studies. First, we implemented a priming paradigm in which context sentences required no task and were presented for a limited amount of time (from now on we will refer to these sentences as “prime sentences”). By using such a priming paradigm we aimed at preventing participants from deliberately drawing upon strategic processing for comprehending prime sentences. Our aim was to rule out the possibility that the involvement of sensory information in language comprehension was the consequence of a late post-lexical strategy to imagine objects and objects properties. Given that recent studies (Trumpp et al., 2013, 2014) showed that subliminally presented sound and action words can activate auditory and motor systems, we reasonably hypothesized to find the MSE although no instructed task was required on prime sentences presented for a limited amount of time. The second key difference is that we used prime sentences that made a linguistic description of the pure perceptual stimuli used in Spence et al. (2001) and in van Dantzig et al. (2008) studies so that to exclude connections between amodal symbols as a possible explanation of the effect. Given that language comprehension involves the construction of a perceptual simulation (Barsalou, 1999; Zwaan, 2004), and that perceptual simulations only involve exogenous attentional mechanisms, it is likely that reading or listening to a linguistic description of a pure perceptual stimulus could pre-activate specific sensory modalities, which could then facilitate or interfere with the processing of subsequent semantic information.

Moreover, in order to avoid any possible semantic association between prime and target sentences, concepts were either semantically unrelated or low semantically related. In Appendix A we report a norming study that we have conducted to assess the semantic relatedness of our stimuli (see also Marques, 2006 on the effects of semantic relatedness). Our participants were first presented with a prime sentence describing a *LIGHT* or a *SOUND*’s perceptual property (e.g., “*LIGHT* is *flickering*”; “*SOUND* is *echoing*”) and then with a target sentence (e.g., “*BUTTER* is *yellowish*”, “*BRUSHWOOD crackle*”) upon which a property verification judgment was to be made.

Finally, we also included a further manipulation by introducing neutral prime stimuli, that is, prime stimuli which did not convey any sensory information. Our purpose was to compare performances on target sentences preceded by sensory information (i.e., visual and auditory prime sentences) with performances on target sentences that were not preceded by sensory information (i.e., neutral prime sentences). Since neutral prime items were not expected to trigger a perceptual simulation, that is, they were not expected to involve any attentional mechanisms which could pre-activate a specific sensory modality, we either predict neither facilitation nor interference due to the fact that participants were unable to pre-activate a sensory modality.

We ran an Experiment in which prime and target sentences conveying both visual and auditory contents were presented either visually or aurally. We predicted to find the MSE even with this modified property verification paradigm. In other words, we expected to find a better performance when prime and target sentences share the same modality compared to when they do not.

## Methods

### Participants

Sixty-four students from the University of Bologna (43 females; mean age: 20.26, SD: 1.58) participated in this study in return for course credits. All participants were Italian native speakers, had normal or corrected-to-normal vision and hearing by self-report, and were naïve as to the purpose of the experiment. Participants were randomly assigned to one of the two between-subjects conditions (visual vs. auditory). The experiment was approved by the Psychology Department’s ethical committee of the University of Bologna. Written informed consent was obtained from all individual participants included in the study. Minors did not take part in this study.

## Materials

### Prime Items

We used 96 prime items. Forty-eight consisted of 24 visual and 24 auditory concept-property pairs. The concepts presented were always “*LIGHT*” and “*SOUND*” and the properties were adjectives associated with them (e.g., “*flickering*/*echoing*”, for the visual and auditory concepts, respectively). Twenty-four properties were used, 12 for the visual and 12 for the auditory prime sentences. These properties were repeated once throughout the experiment. Twenty properties out of 24 were taken from the norming study by Lynott and Connell (2009), who classified several object’s properties along a unimodality – multimodality continuum. The twenty properties we selected from their pool were all unimodal, being mainly perceived either through the sense of sight or through the sense of hearing. Lynott and Connell (2009) found indeed that using unimodal properties instead of multimodal ones leads to a markedly larger MSE. Since our experimental design needed 24 properties, following Lynott and Connell’s (2009) combined criterion, we selected four further properties after 50 Italian adjectives had been rated by 22 participants (see Appendix B). For an overview of the visual and auditory prime sentences see Appendix C.

The other 48 prime items consisted of neutral stimuli, that is, for the visual condition a meaningless strings of symbols (e.g., # ° ˆ ? ^∗^) and for the auditory condition a white noise. Both served to create a neutral modality compared to the same and different ones.

### Target Sentences

We used 96 target sentences: 48 critical target sentences, consisted of half visual and half auditory concept-property pairs taken from the van Dantzig et al.’s (2008) study. In these critical pairs the property was always true of the concept (e.g., “*BUTTER* is *yellowish*”, “*BRUSHWOOD crackles*”). Each pair was used only once in both the visual and auditory condition of the experiment. Two properties were repeated once across the pairs, although paired with different concepts (i.e., “a *BEE buzzes*”, “a *FLY buzzes*”; “*BROCCOLI is green*”, “*SPINACH is green*”). For an overview of the visual and auditory target sentences see Appendix C. The remaining 48 stimuli were filler sentences, always taken from van Dantzig et al. (2008). In the filler sentences the property was always false of the concept. Twelve filler sentences had a false visual property (e.g., “the *WATER is opaque*”), 12 had a false auditory property (e.g., “the *COMB sings*”), whereas the remaining 24 filler sentences had a false property that did not belong to any modality (e.g., “the *BED is sleepy*”). This latter type of fillers was used in order to avoid participants from basing their answers on a superficial word-association strategy, rather than on deeper conceptual-processing (see also Solomon and Barsalou, 2004).

For both the visual and auditory condition, each participant was presented in total with 96 prime sentences (48 modal and 48 neutral) followed by 96 target sentences (48 critical and 48 filler) throughout the experimental session. Prime and target items were randomly combined to form same, different and neutral modality conditions. Each target sentence appeared in the same, different, and neutral modality conditions, counterbalanced across lists. This resulted in a comparable distribution of semantic relatedness and stimulus size measures across experimental conditions. To sum up, the critical targets could be combined with: (1) a neutral prime item (neutral modality); (2) a same-modality prime sentence (visual–visual; auditory–auditory, same modality) or (3) a different-modality prime sentence (visual-auditory, auditory-visual, different modality).

### Procedure

The experiment took place in a dimly lit room. Participants sat in front of a computer screen, at a distance of about 60 cm. For the visual condition, each trial started with the presentation of a fixation cross (0.5 cm × 0.5 cm) for 500 ms. Immediately after the fixation, the prime sentence appeared in the middle of the screen for 1500 ms. Then, the target sentence was displayed on the center of the screen until a response was given or until 3000 ms had elapsed. Prime and target sentences ranged from 5.9 to 17.3 cm (from 9 to 29 characters) which resulted in a visual angle range between 5.6° and 16.5°. All words were bold lowercase Courier new 18. These measures were the same across all conditions. Participants were instructed to read the prime and target sentences and then to judge, as quickly and accurately as possible, whether in each target sentence the property was true of the concept. Participants underwent a short practice session of 24 stimuli (different from those used in the experimental blocks), during which a feedback was given about their response. For the auditory condition, the procedure was the same, except that (1) a “bip” sound was presented in alternative to the fixation cross in order to announce the beginning of a new trial; (2) the prime and the target sentences were presented aurally, through headphones, for 2000 ms and 4000 ms, respectively. In both the visual and the auditory condition, half of the participants pressed the “s” and “k” keys of a “qwerty” keyboard when the property was, respectively, true and false of the concept, that is, when the target was a critical or a filler sentence, respectively. The other half of the participants was assigned to the reverse mapping.

In order to control for sequence effects, we avoided to present the same modality for more than two consequent trials. For example, a prime sentence in the visual modality could be followed by another visual prime sentence only once. Then an auditory or neutral prime had to be shown. The same rule held for the target sentences. Two different sequences, composed of the same 192 concept-property pairs, were built. In both visual and auditory conditions, the sequence presentation was balanced across participants, such that half of the participants was presented with one sequence and the remaining ones with the other.

Participants underwent two blocks of 48 prime sentences followed by 48 target sentences each (24 critical and 24 filler) and could take a short break between them. The experiment lasted ∼ 20 min.

## Results

Responses to filler sentences were discarded. Omissions (3.74%), Incorrect responses (17.90%) and RTs faster/slower than the overall participant mean minus/plus 2 SD (3.61%) were excluded from the analyses.

**FIGURE 1:**
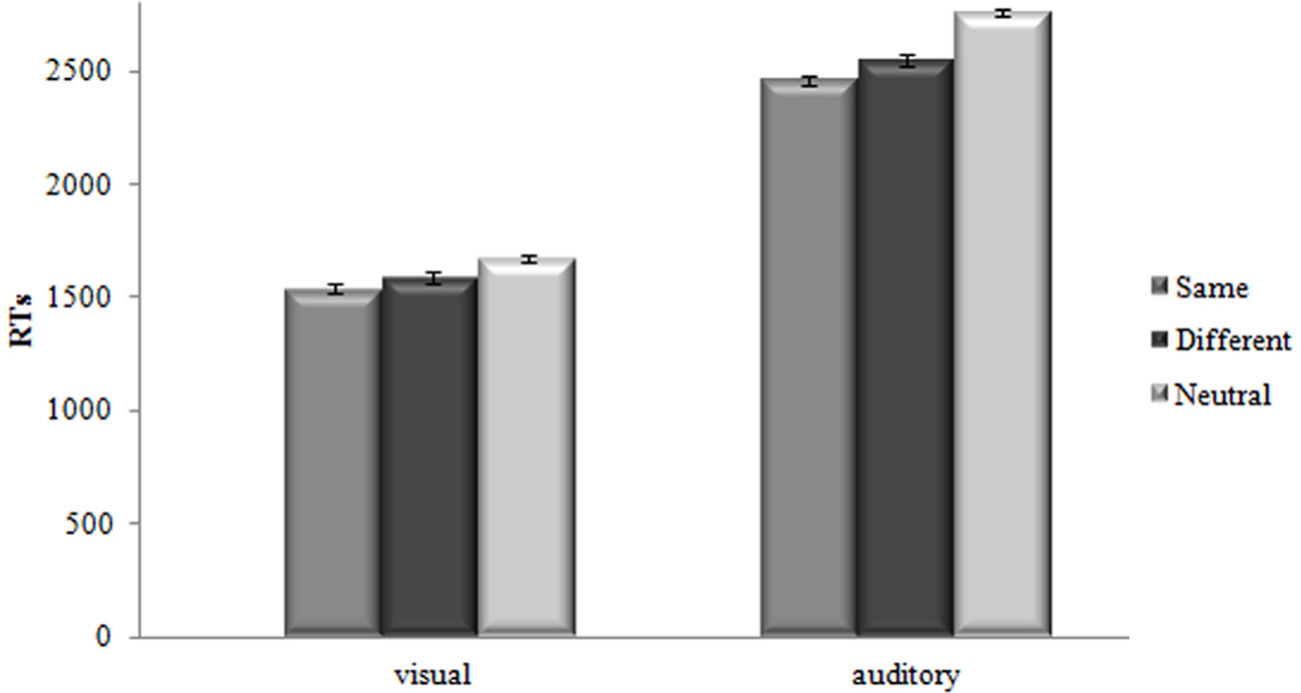
**Mean Response Times (RTs) (in Milliseconds) as a Function of *Modality* (same, different, neutral) for both visual and auditory conditions.** Bars are standard Errors.

Mean Response Times (RTs) of the correct responses were submitted to a Repeated Analysis of Variance (ANOVA) with *Modality* (same vs. different vs. neutral) as the within-subject factor and *Condition* (visual vs. auditory) as the between-subjects factor^3^ (see **Table 1** and **Figure 1** for the results).

**Table 1:** Mean Response Time (RTs) (in Milliseconds) with Standard Deviations in parenthesis, as a Function of Modality (same, different, neutral) for both visual and auditory conditions.

	Visual Condition	Auditory Condition
Same	1538 (178.3)	2462 (202.2)
Different	1588 (206.5)	2552 (245.4)
Neutral	1676 (222.2)	2756 (349.1)
MSE	50^∗^	90^∗^

*The MSE is computed by subtracting RTs in the same modality from RTs in the different modality. Asterisks denote significant differences.*

Results indicated that the main effect of *Modality, F*(2,124) = 58.32, *MSE* = 13302.62, *p* < 0.001, $\eta_{p}^{2}$ = 0.485, was significant. Paired-sample *t*-tests showed that decision latencies for same modality targets (*M* = 2000 ms, *SD* = 502.73 ms) were shorter than for different modality targets (*M* = 2070 ms, *SD* = 535.67 ms) *t*(63) = 5.7, *p* < 0.001, and decision latencies for neutral modality targets (*M* = 2216 ms, *SD* = 616.95 ms) were longer than for both same and different modality targets *t*(63) = 8.1, *p* < 0.001, *t*(63) = -6.4, *p* < 0.001.

The main effect of *Condition, F*(1,62) = 320.32, *MSE* = 146787.41, *p* < 0.001, $\eta_{p}^{2}$ = 0.838, resulted as significant, showing that the auditory condition was slower than the visual one (2590 ms vs. 1600 ms, respectively). However, it is worth mentioning that this result is due to a technical specification in the procedure: aurally presented prime and target sentences lasted longer than the visual ones, considering that spoken sentences need to be listened to until the end before participants could be able to release a response, while visually presented sentences were completely available at once.

The interaction between the *Modality* and *Condition* factors was significant, *F*(1.4,87.1) = 7.88, *MSE* = 18941.26., *p* < 0.001, $\eta_{p}^{2}$ = 0.113.

Paired-sample *t*-tests in the visual condition showed that decision latencies for same modality targets were faster than for different modality targets *t*(31) = 3.2, *p* < 0.01, whereas decision latencies for neutral modality targets were slower than for both same and different modality targets *t*(31) = 6.5, *p* < 0.001, *t*(31) = -4.1, *p* < 0.001. Similarly, paired-sample *t*-tests in the auditory condition showed that decision latencies for same modality targets were faster than for different modality targets *t*(31) = 4.9, *p* < 0.001, whereas decision latencies for neutral modality targets were slower than for both same and different modality targets *t*(31) = 6.5, *p* < 0.001, *t*(31) = -5.4, *p* < 0.001. In order to investigate the difference between the magnitude of the MSEs found, we run an additional Univariate ANOVA with the magnitude of the MSEs as dependent variable and the *Condition* as the only between-subjects factor. The magnitude of MSEs was computed by subtracting the mean RT for the same modality from the mean RT for the different modality. Results showed that the MSE found for the visual condition (50 ms) did not differ from the one found for the auditory one (90 ms), *F*(1, 62) = 2.8, *p* = 0.10, $\eta_{p}^{2}$ = 0.043.

In order to exclude a speed accuracy trade-off, mean of the incorrect responses and omissions were submitted to an ANOVA with the same factors as those of the RTs analysis. As to the incorrect responses, neither the main effects, nor the interaction were significant, *F*_s_ < 2, *p*_s_ > 0.74, $\eta_{p}^{2}$ < 0.004. As to the omissions, results indicated that the main effect of *Modality F*(2,124) = 11.32, *MS*_e_ = 36.22, *p* < 0.001, $\eta_{p}^{2}$ = 0.155 was significant. In addition, the interaction between *Modality* and *Condition* was significant, *F*(2,124) = 4.31, *MS*_e_ = 51.35, *p* < 0.05, $\eta_{p}^{2}$ = 0.065. Paired sample *t*-tests showed that in the visual condition participants made more omissions in the neutral modality (3.7%) than in the different one (1.4%), *t*(31) = 2.5, *p* < 0.05. While in the auditory condition all the comparisons resulted significant showing that participants made less omissions in the same modality (1.3%) than in the different one (3.2%), *t*(31) = 2.3, *p* < 0.05, whereas omissions in the neutral modality (7.6%) outreached omissions in both different and same modalities, *t*(31) = 2.6, *p* < 0.05, *t*(31) = 3.3, *p* < 0.05.

## Discussion

The goal of the current study was to investigate whether the MSE is the result of the activation of sensory information due to exogenous attentional mechanisms. We used a different paradigm from previous studies in order to exclude strategic processing and amodal symbols accounts of concepts as possible explanations of the effect. In line with the hypotheses, our findings showed a robust MSE, that is, a facilitation for the processing of those target sentences the modality of which was formerly primed by a linguistically described perceptual stimulus. In other words, participants were faster when they responded to a target sentence in the same modality as the previous prime sentence rather than different. These results confirm that when the target’s modality correspond to the one pre-activated by the content of the prime sentence, RTs are speeded, while when these modalities do not correspond the time needed to complete the task is slowed down.

It is worth noting that our findings also showed slower RTs and a higher percentage of omissions for the neutral modality compared to the different modality. One might argue that the different modality could be expected to be the slowest modality. Indeed, activating information that does not correspond with what has to be processed later (i.e., different modality) should interfere with the processing of subsequent information and, thus, should require longer RTs overall. However, the slowest performances observed with the neutral modality were possibly due to the fact that in this case the prime items (i.e., meaningless strings of symbols or white noise) were perceptually non-informative. Unlike the visual and auditory prime sentences, the neutral prime did not pre-activate any specific sensory modality, neither correspondent nor non-correspondent. If the account for the MSE and the hypothesis that a neutral prime do not pre-activate any sensory modality are correct, we could assume that the neutral prime did not trigger any perceptual simulation. Since no perceptual simulation took place with neutral prime items, participants could not take advantage of a general activation of the sensory system and this consequently resulted in an overall delay and a higher occurrence of omissions in the processing of the target sentences. This result is in line with a recent study by Connell et al. (2012), in which the conceptual processing of non-manipulable objects (e.g., cars or windmills) was not influenced by either a prior tactile or proprioceptive stimulation, showing that perceptually informative stimuli implied no facilitation effect but rather slowed down the RT needed to complete a task on perceptually non-informative stimuli.

A potential concern is that participants could rely on a word association strategy to perform the property verification task upon target sentences. However, in the current experiment participants could not carry out a superficial processing of stimuli, using only word-level representations, for at least two reasons. First, the semantic domains across prime and target sentences were very distant to allow for a word association strategy (see also Marques, 2006): while target sentences described perceptual properties of objects, prime sentences described properties of two perceptual categories (i.e., light and sound), hence no main semantic association was available across them. Second, in order to avoid participants using the word association strategy, we drew upon highly associated concepts and properties on false trials (i.e., fillers). Indeed, previous studies (Smith et al., 1974; James, 1975; McCloskey and Glucksberg, 1979) showed that manipulating the difficulty of false trials varies the depth of processing on true trials (see also Solomon and Barsalou, 2004). Therefore, rather than have participants reject unassociated false properties for concepts in the filler trials (e.g., *unripe* for *BED*), we had participants reject associated ones (e.g., *sleepy* for *BED*). For associated false properties, participants could not respond “false” on the basis of the word association strategy because the concept and the property were actually somehow associated (i.e., sleepy people go to bed). Thus, in order to determine whether the property was true of the concept, participants must access conceptual knowledge for *BED* and *sleepy* and realize, for instance, that rather than being sleepy a bed is used by sleepy people.

Overall, the results of the present study boost and broaden previous findings which showed a significant MSE during an on-line perceptual task (Spence et al., 2001), a property verification task (Pecher et al., 2003) and across perceptual and conceptual tasks (van Dantzig et al., 2008). More broadly, our results support the accounts of the role of perceptual attention on conceptual processing (Connell and Lynott, 2010, 2012, 2014) showing that exogenous attentional mechanisms are at work during perceptual simulation and are responsible for the MSE. Although we cannot completely rule out that the MSE we found is due to strategic or imagery processes, the use of a standard priming paradigm represents an important difference compared to previous work. Indeed, while in previous studies the sensory modality was likely to be strategically activated when performing the task on the context sentence, in our experiment we found a MSE even though participants were not required to perform any task on the prime sentences. That is, in our experiment it was completely unnecessary to directly and explicitly pre-activate a specific sensory modality, therefore the MSE we found is likely to be due to an implicit and indirect pre-activation of sensory modalities. Ultimately, we showed that the MSE also occurs when participants are prevented from drawing upon strategic processing, furthering the hypothesis that the MSE arises from a simulation process during which exogenous attention operates. In addition, we showed that not only a perceptual stimulus (van Dantzig et al., 2008) but also a perceptual linguistically described stimulus triggers the pre-activation of a sensory modality: reading or listening to a sentence describing a light or a sound’s perceptual property sufficed to spark off a simulation, even though no task was required on that sentence.

## Conclusion

The simulation of an object varies considerably across occasions. When reading or listening to a sentence involving a particular object in a certain situation, implicit perceptual attention (i.e., exogenous attention) activates a specific modality. If that modality had been previously activated by either a perceptual stimulus or a perceptual linguistically described stimulus, the processing of semantic information that relates to that modality in the sentence is facilitated. This is far from implying that any given object does only relate to a certain modality. Rather, other relevant modalities might be temporarily inhibited. In facts, modalities represented in simulations vary on the basis of their activation. Future exploration of the MSE could use this modified property verification paradigm with multimodal concepts in order to investigate what happens when multiple modalities compete during a simulation.

## Author Contributons

All the authors conceived and designed the experiment.

Performed the experiments: ES

Analyzed the data: ES, GB, LL

Drafted the paper: ES, GB, LL

All the authors provided critical revisions.

## Conflict of Interest Statement

The authors declare that the research was conducted in the absence of any commercial or financial relationships that could be construed as a potential conflict of interest.

## Footnotes

1 We use uppercase italics for concepts (*VIOLIN*) and lowercase italics for properties of the concepts (*melody*) throughout the whole manuscript.

2 Only a *too slow* feedback was provided when participants’ responses were slower than 2 s.

3 In order to control for items variability, we conducted a Univariate ANOVA with *Target* (i.e., critical concept-property pairs) as a fixed factor and *Modality* as a random factor. Crucially, for both the visual and auditory conditions, the *Target* x *Modality* interaction was not significant, *F*_s_ < 1.1, *p*_s_ > 0.28, $\eta_{p}^{2}$ < 0.062. Since these results ruled out the possibility that the effect was due to some particular target among the others, we decided to report and discuss the ANOVA in the main text.
