# Supplementary material for: The modality-switch effect: visually and aurally presented prime sentences activate our senses
Source: Front Psychol. 2015 Oct 30;6:1668. doi: 10.3389/fpsyg.2015.01668 (PMC4627474; doi:10.3389/fpsyg.2015.01668)
Supplement: Supplementary file 1 [file Data_Sheet_1.DOCX]

***Supplementary Material***

**The modality-switch effect: Visually and aurally presented prime sentences automatically activate our senses**

**Elisa Scerrati*, Giulia Baroni, Anna Maria Borghi, Renata Galatolo, Luisa Lugli, Roberto Nicoletti**

*** Correspondence:** Anna Maria Borghi: [annamaria.borghi@unibo.it](mailto:annamaria.borghi@unibo.it)

Elisa Scerrati: [elisa.scerrati@unibo.it](mailto:elisa.scerrati@unibo.it)

**Appendix A**

**Norming study**

We ran a norming study in which sixteen new participants (6 males; mean age: 22.62, SD: 4.36) were required to read each of the 12 visual and 12 auditory prime sentences and to report the first five words that came to mind in a free association task. We hypothesized that if the prime and target sentences used in our experiment were semantically related, participants should report our target words and, more specifically, they should report our target words in the first positions. Results showed that 33 words out of the 101 actually used in our target sentences were reported. Furthermore, we observed that these 33 words occurred 154 times across subjects considering all the five positions (154*100 /1920 (16 participant * 24 prime sentences * 5 positions) = 8%), and crucially 31 times considering only the first position (31*100/384 (16 participant * 24 prime sentences) = 8%). For example the word “red” was reported 10 times, but only 3 times in the first position. Considering these results, we concluded that our prime and target sentences were either semantically unrelated or low associated.”

**Appendix B**

**Rating of the 50 Italian adjectives**

A set of 50 words was selected from the Italian dictionary Sabatini-Coletti. Each word (either an adjective or present participle of a verb) could belong to the auditory or to the visual sensory modality (e.g., ritmato, accecante).

The rating was administered with an on line procedure. Twenty-two participants, all Italians native speakers (13 females; mean age: 23 years old; sd. 4 years) were tested. To avoid order presentation effects, participants were split into two equal groups having two different orders of item presentation. Participants’ task was to rate, on 5 separate 5-points Likert scales (where 1 = not at all; 5 = greatly) the extent to which each item is experienced through each of the five senses. For instance, participants were faced with the item “bright” and were asked to rate to what extent they experienced this property by the touch, the hearing, the sight, the sense of smell and by the taste.

Participants’ average score for each item in each modality was computed. In order to compute the modality exclusivity and modality strength indexes as in Lynott and Connell (2009) and compare our 5-points (1-5) Likert scale to their 6-point (0-5) scale we applied the following conversion formula: (5/4* the score obtained for the item in each modality) – 5/4. This allowed us to keep the threshold of the modality strength and the modality exclusivity to 3.5 and .65 respectively. Four additional items (2 visual, 2 auditory) which scored strong on modality strength (3.5-5) and high on modality exclusivity (65%-100%) were selected.

**Appendix C**

**Overview of the visual and auditory prime and target sentences**

| **Visual Prime Stimuli** | **Visual Prime Stimuli** | **Visual Target Stimuli** | **Visual Target Stimuli** |
| --- | --- | --- | --- |
| *English version* | *Italian translation* | *English version* | *Italian translation* |
| Light is bright | La luce è intensa | Butter is yellowish | Il burro è giallognolo |
| Light is colourful | La luce è colorata | Broccoli is green | Il broccolo è verde |
| Light is dazzling | La luce è abbagliante | Chocolate is dark brown | Il cioccolato è marrone |
| Light is dim | La luce è soffusa | An eggplant is dark purple | La melanzana è viola |
| Light is flickering | La luce è intermittente | An inner tube is black | La camera d'aria è scura |
| Light is gleaming | La luce è splendente | A cassette tape is black | La musicassetta è nera |
| Light is glowing | La luce è raggiante | A diamond glistens | Il diamante brilla |
| Light is gold | La luce è dorata | A squirrel is red-brown | Lo scoiattolo è rossiccio |
| Light is shimmering | La luce è scintillante | An ice cube is transparent | Il cubetto di ghiaccio è trasparente |
| Light is translucent | La luce è luminosa | A cellar is dark | La cantina è buia |
| Light is blinding | La luce è accecante | A jellyfish is translucent | La medusa è lucida |
| Light is flashing | La luce è lampeggiante | A leopard is spotted | Il leopardo è maculato |
|  |  | An orca is black-and-white | L'orca è bianca e nera |
|  |  | Peppermint is white | La mentina è bianca |
|  |  | A chessboard is chequered | La scacchiera è a quadri |
|  |  | A razorblade is silver | La lametta è argentata |
|  |  | A tennis ball is yellow | La palla da tennis è gialla |
|  |  | A walnut is brown | La noce è bruna |
|  |  | A wasp is striped | La vespa è striata |
|  |  | A swimming pool is azure blue | La piscina è azzurra |
|  |  | Ham is pink | Il prosciutto cotto è rosa |
|  |  | Honey is golden-yellow | Il miele è ambrato |
|  |  | Mayonnaise is light yellow | La maionese è giallina |
|  |  | Spinach is dark green | Gli spinaci sono verdi |
| **Auditory Prime Stimuli** | **Auditory Prime Stimuli** | **Auditory Target Stimuli** | **Auditory Target Stimuli** |
| *English version* | *Italian translation* | *English version* | *Italian translation* |
| Sound is deafening | Il suono è assordante | A bee buzzes | L'ape ronza |
| Sound is echoing | Il suono è echeggiante | A flute is high-pitched | Il flauto è di tono acuto |
| Sound is hushed | Il suono è sommesso | A scooter hums | Il motorino strepita |
| Sound is loud | Il suono è alto | A bicycle bell rings | Il campanello trilla |
| Sound is mute | Il suono è muto | A church organ clangs | L'organo vibra |
| Sound is noisy | Il suono è rumoroso | A cricket chirps | Il grillo canta |
| Sound is shrill | Il suono è penetrante | A saxophone blares | Il sassofono è squillante |
| Sound is sonorous | Il suono è altisonante | A ship’s horn is low-pitched | Il fischio della nave è basso |
| Sound is squealing | Il suono è stridente | A siren wails | La sirena urla |
| Sound is husky | Il suono è rauco | A station hall is noisy | La stazione è chiassosa |
| Sound is croaking | Il suono è gracchiante | A tram grinds | Il tram sferraglia |
| Sound is audible | Il suono è udibile | A triangle jingles | Il triangolo tintinna |
|  |  | A trumpet sounds shrill | La tromba è stridula |
|  |  | A typewriter rattles | La macchina da scrivere ticchetta |
|  |  | A fly buzzes | La mosca ronza |
|  |  | An airplane is loud | L'aereo è roboante |
|  |  | A truck honks | L'autocarro strombazza |
|  |  | An alarm clock ticks | L'orologio fa tic tac |
|  |  | Autumn leaves rustle | Le foglie frusciano |
|  |  | Brushwood crackles | Il sottobosco crepita |
|  |  | High heels tap | I tacchi alti picchiettano |
|  |  | Thunder rumbles | Il tuono rimbomba |
|  |  | Pans clang | Le pentole urtano fragorosamente |
|  |  | A railroad crossing rings | Il passaggio a livello suona |
